# A Comparative Study of Genital Lichen Sclerosus Transcriptomes

**DOI:** 10.32607/actanaturae.27889

**Published:** 2026

**Authors:** S. D. Margasyuk, A. L. Kuznetsova, D. A. Skvortsov, E. M. Alekberov, M. M. Iritsyan, S. A. Pulbere, S. V. Kotov, A. A. Sokolova, D. D. Pervouchine

**Affiliations:** Center for Molecular and Cellular Biology, Moscow, 121205 Russia; Faculty of Chemistry, Moscow State University, Moscow, 119991 Russia; N.I. Pirogov Russian National Research Medical University, Moscow, 117513 Russia; N.I. Pirogov City Clinical Hospital No. 1 of the Moscow Department of Health, Moscow, 119049 Russia; Yevdokimov Moscow State University of Medicine and Dentistry, Moscow, 127473 Russia

**Keywords:** Genital lichen sclerosus, GLS, inflammation, innate immunity, transcriptomics, long non-coding RNA

## Abstract

Genital lichen sclerosus (GLS) is a chronic inflammatory dermatosis that affects the genital skin. Despite different clinical manifestations, the pathogenesis of GLS in men and women is believed to be common and is attributed to a combination of autoimmune and genetic factors. In this study, we compared the transcriptomic profiles of penile (mGLS) and vulvar lichen sclerosus (VLS), aiming to identify commonly deregulated genes. We observed a substantial heterogeneity in the transcriptomic signatures in mGLS samples, which is driven by different compositions of immune infiltrates. In mGLS, gene expression signatures strongly indicate epidermis dysfunction and overexpression of the epithelial inflammation marker Keratin 6 (KRT6) and chitinase CHIT1. No significant changes in the expression levels of known GLS markers, such as VIM, CTNNB1, LGALS7 and ECM1, were detected. However, significant changes in the expression levels of the genes associated with autoimmune diseases and the genes upregulated in squamous cell carcinoma, including TNF, CCNB1 and RUNX3, were observed. There was no enrichment in the polyU/UC insertions that were reported previously. Instead, we have identified a long non-coding RNA DRAIC with a high coding potential that is commonly upregulated in mGLS and VLS. Taken together, our results provide a comprehensive picture of the shared transcriptomic signatures, including novel biomarkers and potential therapeutic targets.

## INTRODUCTION

Genital lichen sclerosus (GLS) is a chronic inflammatory disease affecting genital skin in men and women [1]. It manifests itself as white atrophic patches in the affected area, often accompanied by itching, soreness, and painful urination. GLS is characterized by a persistent, recurrent course often leading to severe complications such as cicatricial phimosis, paraphimosis, and urethral stenosis in men [2], and urinary obstruction, vulvar ostium stenosis, and degeneration of genital tissues in women [3]. Furthermore, individuals with GLS of both sexes are at an increased risk of developing squamous cell carcinoma [4].

In spite of different clinical presentations, the underlying causes of GLS in the two sexes are commonly attributed to a combination of autoimmune and genetic factors, as well as other conditions, such as skin damage or chronic contact with urine [5]. Several molecules have been identified as being associated with the immunopathology of GLS. Among those are the product of the ECM1 gene: a 85-kDa secreted glycoprotein that is expressed in different splice variants, with autoantibodies frequently elevated in GLS patients [6, 7]; microRNA miR-155 known to contribute to sclerotic tissue formation; galectin-7, a pro-apoptotic keratinocyte protein promoting fibroblast proliferation, and others [8, 9]. HLA class II genotypes, particularly HLA-DQ7, are frequently associated with GLS in both sexes, with more than half of affected females carrying this haplotype [10, 11]. Comorbidity studies indicate that GLS patients frequently have at least one other autoimmune disease [12, 13]. Yet, the molecular causes of GLS remain largely unknown and no universal biomarker currently exists for its definitive diagnosis.

**Table 1:** mGLS and VLS transcriptome profiling experiments including bulk and single-cell RNA-seq and DNA microarrays

GLS/Method	Bulk RNA-seq	Single-cell	RNA-seq MicroArray
mGLS	Host-microbe interactions upon dysbiosis of tissue microbiota [15]	Fibroblast-mediated pathogenesis [16]; Characteristic subset of cells including fibroblasts [18]; Epigenetic analysis [19]	Gene expression profiling [20]; Gene expression patterns in Congenital phimosis [21]
VLS	Transcriptome profiling and network analysis [22]; HCV poly U/UC sequence-induced inflammation [14]	Keratinocytes as key players in the pathogenesis of VLS [23]	Autoimmune phenotype [24]

A number of studies approached the task of determining the mechanism of GLS pathogenesis by transcriptome profiling, such as bulk and single-cell RNA sequencing (RNA-seq) integrated with multi-omics approaches, and also by earlier techniques such as DNA microarrays (Table 1). These efforts generated a number of challenging hypotheses; for instance, on a correlation with an abnormal antivirus response due to the presence of Hepatitis C Virus poly U/UC sequences in vulvar lichen sclerosis (VLS) [14], on the host–microbe interactions upon dysbiosis of tissue microbiota in male genital lichen sclerosus (mGLS) [15], and on the role of the crosstalk between fibroblasts and T cells in fibroblast-mediated pathogenesis in the latter [16]. While GLS in males and females is often considered as one etiology [17], their shared underlying molecular causes remain unknown and, furthermore, no comparative study of VLS and mGLS has been conducted. In revisiting this problem, we performed high-throughput transcriptome profiling of biopsy samples from the genital sites of mGLS patients and healthy donors by RNA-Seq in order to compare the transcriptomic signatures of VLS and mGLS, with the aim to identify commonly deregulated genes.

## EXPERIMENTAL

**Biopsy and sample collection**

The clinical study was approved by the Institutional Review Board of the Skolkovo Institute of Science and Technology and by the Ethics Committee of the N.I. Pirogov Moscow City Clinical Hospital No. 1. In the study, ten male patients aged between 18 and 42 years (median age 28 years) were enrolled at the Day Hospital of the Urology Division of N.I. Pirogov Moscow City Clinical Hospital No. 1. The GLS group consisted of five patients with a clinically confirmed diagnosis of genital lichen sclerosus. The control group consisted of five healthy individuals undergoing planned surgical treatment. The operating urologist marked three areas of the sclerotically altered foreskin in each patient from the GLS group and three unaffected areas of the foreskin in each patient from the control group, each area comprising a square sized 0.5 × 0.5 cm. Upon dissection, foreskin fragments were minced with a scalpel, transferred into a cryovial, and stored in liquid nitrogen at –80°C (collection time ranged from 50 to 85 s; median collection time was 65 s). The remaining biomaterial was fixed in 10% buffered neutral formalin, compactified, and embedded in paraffin. The 4-μm-thick sections of the resulting paraffin blocks were prepared on the microtome, stained with hematoxylin and eosin, and subjected to a histological examination.

**RNA extraction**

RNA extraction and isolation were performed on frozen tissue samples (10–30 mg) using a Purelink mini kit (ThermoFisher, USA). The frozen tissue samples were added into CK14 tubes precooled to 4°C containing 0.6 mL of the lysis buffer (ThermoFisher) and beads (Bertin Technologies). Precellys®24 was applied three times for 40 s at 6,300 rpm with a 1-min interval. The tubes were placed on ice between each cycle. Each sample was centrifuged at 12,000 g for 2 min; the extract was transferred to a new tube, and 600 μL of 70% ethanol was added. The mixture was vortexed, and 600 μL of the mixture was transferred to a spin cartridge to centrifuge at 12,000 g for 15 s. The flowthrough was discarded; 600 μL of the mixture was added into the spin cartridge again and centrifuged at 12,000 g for 15 s. The cartridge was washed three times by adding 700 μL, 500 μL, and 500 μL of the Wash Buffer I, respectively, followed by centrifuging at 12,000 g for 15 s. The cartridge was placed into a  new tube; 100 μL of RNase-free water was added into its center, followed by incubation for 1 min and centrifuging at 12,000 g for 2 min at room temperature. The RNA concentration was measured by photometry, with a 260/280 nm ratio of optical density in the 2.0–2.1 range. The RNA integrity score (RIN) was measured using an Agilent 2100 Bioanalyzer on samples containing on average 128 ng/μL RNA, yielding the median RIN score of 7. Two samples (mGLS sample 7 and control sample 8) with a RIN score below 6 were discarded. The remaining four mGLS samples and four control samples were chosen for library preparation and RNA-seq.

**RNA-seq libraries and sequencing**

PolyA+ RNA libraries were prepared using a NEBNext Ultra II Directional RNA Library Prep Kit for Illumina with Purification Beads (E7765 L, New England Biolabs) and a NEBNext® Magnetic Bead Poly(A)+ mRNA Isolation Module (E7490 L, New England Biolabs) in accordance with the manufacturer’s protocol. NGS was performed, and 150-base single-end reads were collected on an Illumina 2000 sequencing system.

**cDNA synthesis**

One microgram of total RNA was first subjected to RNase-free DNase I digestion (Thermo Fisher Scientific) at 37°C for 30 min to remove contaminating genomic DNA. Next, 500 ng of total RNA was used for complementary DNA (cDNA) synthesis using a Magnus First Strand cDNA Synthesis Kit (Evrogen) for reverse transcription-quantitative PCR (RT-qPCR) to a final volume of 20 μL. cDNA was diluted 1 : 5 with nuclease-free water for quantitative PCR (qPCR).

**RT-qPCR**

qPCR reactions were run in triplicates in a final volume of 12 μL in 96-well plates with 420 nM gene-specific primers and 2 μL of cDNA using a 5XqPCRmix-HS SYBR reaction mix (Evrogen). Primers for qPCR are listed in Supplementary 1, Table S1. A sample without a reverse transcriptase enzyme was included as a control to verify the absence of genomic DNA contamination. Amplification of the targets was carried out on a CFX96 RealTime System (Bio-Rad), with the following parameters: 95°C for 5 min, followed by 39 cycles at 95°C for 20 s, 60°C for 20 s and 72°C for 20 s, ending at 72°C for 5 min. For each primer pair in the PCR analysis, primer efficiency was assessed using a calibration curve, with primer efficiency exceeding 90% in all cases. Changes in gene expression were calculated using the double normalization method (“ddCt”), taking into account PCR efficiency. The GAPDH gene was used as an internal control to normalize gene expression levels.

**Read alignment and transcript quantification**

Adapter trimming and short read filtering were performed using the fastp v0.20 utility [25] with parameters enabling low-quality bases removal ‘-l 35 --cut_ front --cut_right’. Reads were then aligned to the GRCh38 human reference genome using GENCODE v47 transcriptome annotation with STAR v2.7.8a [26]. The following parameters were used: --outFilterMultimapNmax 20 --alignSJoverhangMin 8 --alignSJDBoverhangMin 1 --outFilterMismatchNmax 999 --outFilterMismatchNoverReadLmax 0.04 --alignIntronMin 20 --alignIntronMax 1000000 --alignMatesGapMax 1000000 --chimSegmentMin 15. The gene-level counts were generated by assigning the aligned reads to the annotated features using the featureCounts program from the subread package v2.1.1 [27]. Read counts were normalized as TPM using the rnanorm v2.1.0 package. The quantiseq deconvolution method implemented in the immunedeconv package v2.1.0 was used to estimate the fraction of immune cells in a sample [28, 29].

**Differential gene expression and splicing analysis**

Differential gene expression analysis was performed on the raw gene count matrix using the DESeq2 package (pydeseq2 v0.5.0 [30]). In both the mGLS and VLS datasets, the GLS samples were compared to tissue samples from healthy donors. The PCA analysis was performed on the VSTtransformed gene counts from DESeq. Differential gene expression analysis of TCGA-LUSC paired samples was performed with limma package v3.66 [31]. Genes with adjusted P values below 0.05 and absolute log2 fold change greater than 1 were considered as differentially expressed. The sets of differentially expressed genes were submitted to the DAVID web server for functional enrichment analysis [32]. The coding potential of differentially expressed non-coding RNAs was assessed using transdecoder utility v5.7.1. Differential splicing analysis between the disease and control groups was conducted using the rMATS v4.3.0 software [33]. The downstream analysis focused on the exon skipping events, and only the exons with a median read coverage of 40 reads in at least one sample group were considered. The events with an adjusted P value of less than 0.05 and an absolute inclusion level difference greater than 0.05 were considered as differentially spliced.

**Identification of potential neoantigens**

The potential presence of microbial and viral sequences was assessed by metagenomic analysis of the RNA-Seq data using the Kraken2 v2.1.6 software [34] against the standard database with the default settings. Insertions in reads were quantified using a custom script that extracts the insertion segments from CIGAR strings in BAM files with the corresponding read sequences and alignment coordinates. Insertions containing three or more consecutive uridine nucleotides were counted. The insertions in coding exons were counted separately.

**Statistical procedures**

The data were analyzed using the python version 3.8.2 and R statistics software version 3.6.3. The Benjamini–Hochberg correction was used to account for multiple hypotheses testing and compute the adjusted P values. Throughout the paper, r and P denote the Pearson correlation coefficient and the adjusted P value, respectively; FC denotes the fold change in the gene expression level. Non-parametric tests were performed using normal approximation with continuity correction. In all the figures, the significance levels 0.05 and 0.01 are denoted by ^*^ and ^**^, respectively.

## RESULTS

**Heterogeneity of transcriptomic signatures in mGLS**

**Fig. 1:**
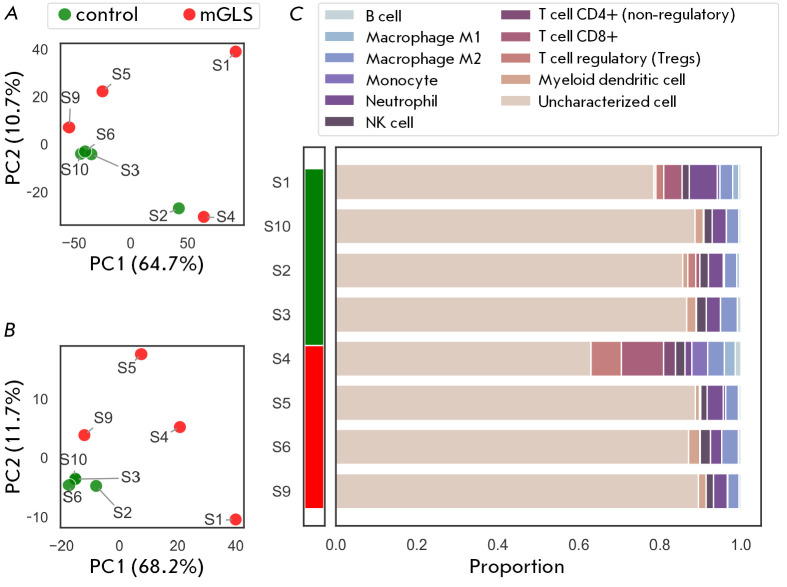
Heterogeneity of transcriptomic signatures in mGLS. (A) Principal component analysis (PCA) of the expression levels of protein-coding genes (control group: S2, S3, S6, S10; mGLS group: S1, S4, S5, S9). (B) PCA of differentially expressed protein-coding genes. (C) Proportion of immune cells in mGLS samples estimated by bulk RNA-Seq deconvolution

High-throughput transcriptome profiling of biopsy samples from the genital skin of four mGLS patients and four healthy donors by RNA-Seq yielded a total of 289 Mln short reads, on average 36 Mln short reads per sample. Gene expression levels were computed from short read counts followed by normalization using the DESeq2 package (Supplementary 2). Principal component analysis (PCA) of gene expression values revealed the lack of clustering by the mGLS vs. control group. Instead, we observed a high degree of heterogeneity within the mGLS cohort, with samples scattering widely along the principal components (Fig. 1A). This was particularly evident along the first principal component (PC1) representing 65% of the variance, where samples formed two distinct subgroups, with samples 1 and 4 being clearly separated from samples 5 and 9. Even when PCA was confined to only differentially expressed genes, the variability of mGLS samples persisted in spite of a clear separation between the mGLS and control groups (Fig. 1B). The scattering of mGLS samples along the principal components accounting for 80% of the variance (PC1 and PC2, 68 and 12%, respectively) suggests the existence of a confounding factor influencing the global gene expression profiles.

Differences in cellular composition, specifically in immune cell infiltration into the diseased tissue, could be a factor contributing to the observed heterogeneity of mGLS samples. To test this hypothesis, we inferred the relative proportions of immune cell subtypes within each sample using a computational deconvolution approach, quanTIseq [28]. It revealed a larger and more variable immune cell infiltration pattern in the mGLS group and only moderate infiltration in the control group (Fig. 1C). Consistent with separate positioning in the PCA plot, samples 1 and 4 exhibited an elevated immune cell proportion and a distinct composition of the immune infiltrate. Sample 4 was characterized by a high relative abundance of CD8^+^ T cells (10.4%) and T-regulatory cells (7.6%), suggesting active immune response under immunosuppression [35, 36]. In contrast, the immune cell fraction in sample 1 was dominated by neutrophils (6.9%), which may be indicative of a different inflammatory microenvironment [37]. This departure from the remaining two mGLS samples, which exhibited relatively low levels of immune cell infiltration, likely drives the variation observed in the PCA.

**Gene expression signatures indicate epidermis dysfunction in mGLS**

**Fig. 2:**
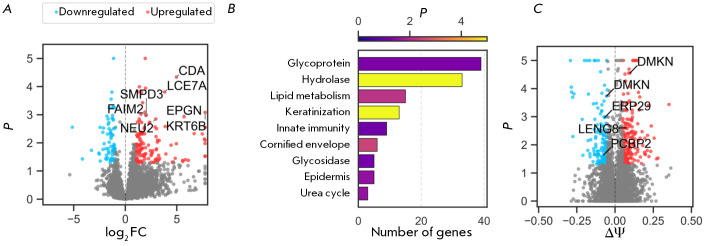
The mGLS transcriptomic profile. (A) The volcano plot of gene expression. Significantly deregulated genes (P < 0.05 and |log2 FC| > 1) are shown in color. (B) Gene set enrichment analysis of upregulated genes. Gene Ontology (GO) categories with P < 0.1 are shown. (C) The volcano plot of differentially spliced cassette exons. Significantly deregulated events (P < 0.05 and |ΔΨ| > 0.05) are shown in color

A comparison of the transcriptomic profiles of human protein-coding genes using the DESeq2 package identified 249 differentially expressed genes (DEGs) between mGLS and the control samples (P < 0.05 and |log_2_ FC| < 1). Of those, 164 were upregulated and 85 were downregulated (Fig. 2A). Functional enrichment analysis of the upregulated genes using the DAVID tool [38] indicated that the primary transcriptional alteration in mGLS affects epidermal integrity and function. The most significantly enriched Gene Ontology (GO) terms and UniProt keywords (KW) were related to epidermal biology (Fig. 2B). The enriched categories included “keratinization” (GO:0031424, P < 10^-10^), “cornified envelope” (GO:0001533, P = 0.003), and “epidermis” (GO:0030280, P  =  0.03). The transcriptomic data also contained detectable immune (“innate immunity”, KW-0399, P =  0.078) and metabolic signatures (“urea cycle”, KW-0835, P = 0.027).

In addition to the analysis of gene expression signatures, we performed differential analysis of alternative splicing by estimating exon inclusion ratios (Ψ, PSI, percent-spiced-in) defined as the number of split reads supporting exon inclusion as a fraction of the combined number of split reads supporting exon inclusion and skipping. A number of exons underwent significant splicing changes in mGLS as evidenced by the change in the exon inclusion ratio (ΔΨ), remarkably in some genes associated with autoimmune disorders (Fig. 2C). Among them were two exons in the dermokine (DMKN) gene, which encodes a skin-specific secreted glycoprotein [39, 40] implicated in the inflammatory bowel disease [41], an exon in the leukocyte receptor gene (LENG8), and an  exon in the poly(C)-binding protein PCBP2, both of which play a role in regulating the T-cell function [42, 43]. All the differentially spliced exons are listed in Supplementary 3.

**Shared transcriptomic signatures in mGLS and VLS**

To uncover the common molecular mechanisms underlying the pathophysiology of GLS in the two sexes, we compared the transcriptomic profiles obtained for the male cohort with the transcriptomic profiles of VLS from a publicly available dataset [14]. The female cohort consisted of nine matched pairs (tissues affected by VLS and adjacent healthy tissues) from the same donors and an additional group from four healthy donors. To ensure a comparable analysis, we identified DEG harbored in autosomes by comparing patient-derived VLS samples to those from healthy donors. This combined dataset allowed us to conduct a cross-sex comparison of the GLS transcriptomes.

The comparison conducted separately in the two sexes revealed a notable disparity in the scale of transcriptomic changes. In the male cohort, we identified 249 DEGs, with 164 being upregulated and 85 being downregulated. By contrast, the female cohort exhibited more extensive deregulation, with 1,116 significantly upregulated and 1,112 downregulated genes. This difference in both the magnitude and balance of DEGs may be attributable to the larger size and, hence, higher statistical power in the female dataset, as well as to biological differences such as a larger and compositionally distinct immune infiltrate in the male cohort.

**Fig. 3:**
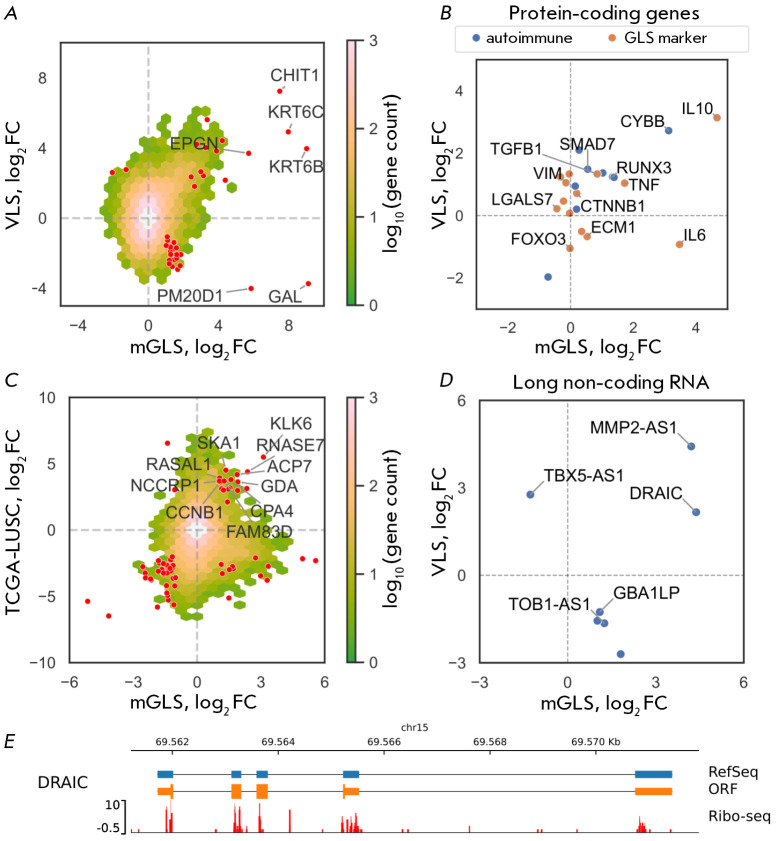
Shared transcriptomic signatures in mGLS and VLS. (A) Density plot of log2 protein-coding gene expression fold changes (log2 FC, GLS vs. control) in mGLS and VLS. The color map displays log10 of the number of genes in each bin. Genes significantly deregulated (P < 0.05 and |log2 FC| > 1) in both mGLS and VLS are indicated by red dots. (B) Expression levels of protein-coding genes with known links to GLS or autoimmune diseases (Table 2). (C) The density plot of protein-coding gene expression fold changes in mGLS and TCGA-LUSC (GLS vs. control and tumor vs. control, respectively). Genes significantly deregulated in both datasets are indicated by red dots. (D) Same as (A) for long non-coding RNAs (lncRNA). (E) Genomic organization of the DRAIC lncRNA including the annotated transcript (RefSeq), the predicted open reading frame (ORF), and aggregated Ribo-seq signal from GWIPS-viz (Ribo-seq)

The expression levels of protein-coding genes (Fig. 3A) showed a weak but significant positive correlation between mGLS and VLS (r = 0.21, P < 10-10), indicating that the transcriptional changes in these diseases are not identical, but that pathogenic alterations or their downstream effects may be similar. One of the genes that appeared consistently upregulated in both mGLS and VLS was Keratin 6 (KRT6), a marker of hyperproliferative and wounded skin states, such as psoriasis [44]. Its shared overexpression aligns with the characteristic manifestations of lesion formation in lichen sclerosus patients. A shared overexpression was also detected for the CHIT1 gene, which codes for the enzyme chitinase 1 and is not directly linked to GLS; however, other members of the chitinase family are known as biomarkers in immunemediated diseases [45].

**Table 2:** The log_2_ FC values for the genes with known links to GLS or autoimmune diseases

ENSEMBL gene ID	Gene name	Description	mGLS	VLS
ENSG00000108821	COL1A1	Collagen type I alpha 1 chain	-0.04	1.33
ENSG00000168542	COL3A1	Collagen type III alpha 1 chain	-0.32	1.24
ENSG00000130635	COL5A1	Collagen type V alpha 1 chain	-0.23	0.46
ENSG00000143369	ECM1	Extracellular matrix protein 1	0.53	-0.67
ENSG00000118689	FOXO3	Forkhead box O3	-0.02	-1.05^**^
ENSG00000136634	IL10	Interleukin 10	4.67^*^	3.14^*^
ENSG00000136244	IL6	Interleukin 6	3.47^*^	-0.93
ENSG00000178934	LGALS7B	Galectin 7B	0.35	-0.51
ENSG00000105329	TGFB1	Transforming growth factor β 1	0.85	1.34
ENSG00000232810	TNF	Tumor necrosis factor	1.72	1.04
ENSG00000141510	TP53	Tumor protein p53	-0.03	0.08
ENSG00000168036	CTNNB1	Catenin β 1	0.2	0.72
ENSG00000026025	VIM	Vimentin	-0.15	1.05^*^
ENSG00000165168	CYBB	Cytochrome b-245 β-chain	3.12^*^	2.72^**^
ENSG00000120738	EGR1	Early growth response 1	-0.72	-1.97
ENSG00000179348	GATA2	GATA binding protein 2	0.15	0.95
ENSG00000183019	MCEMP1	Mast cell expressed membrane protein 1	0.27	2.1
ENSG00000105835	NAMPT	Nicotinamide phosphoribosyltransferase	0.19	0.21
ENSG00000020633	RUNX3	RUNX family transcription factor 3	1.34	1.24
ENSG00000143546	S100A8	S100 calcium binding protein A8	1.03	1.37
ENSG00000163220	S100A9	S100 calcium binding protein A9	1.39	1.23
ENSG00000101665	SMAD7	SMAD family member 7	0.55	1.49^**^

Note. Significant deviations (according to FDR) at the significance level of 5 and 1% are denoted by ^*^ and ^**^, respectively.

Next, we selected genes with known links to GLS or autoimmune diseases (Table 2), including vimentin (VIM) and beta-catenin (CTNNB1), two genes previously identified as histological markers in mGLS, thirteen genes involved in GLS pathogenesis as listed in ref. [17], and nine genes known to be deregulated in at least two autoimmune conditions [46] (Fig. 3B). Contrary to expectations, the VIM and CTNNB1 levels were not elevated at the transcript level in mGLS, while in VLS their expression levels were slightly elevated. Within the set of thirteen GLS-associated genes, we examined two key candidates: the proposed autoantigen ECM1 that was previously reported to be downregulated specifically in mGLS [20] and galectin-7 (LGALS7), but neither of them showed significant deregulation. However, several immunity-related genes were significantly altered. Tumor necrosis factor-alpha (TNF) was non-significantly upregulated in both mGLS and VLS, while interleukin-6 (IL-6) was upregulated specifically in the male patients. Elevation of these pro-inflammatory cytokines is consistent with the activation of an inflammatory response in GLS. For the group of genes associated with the progression of autoimmune diseases, we found significant upregulation of cytochrome B β-chain (CYBB) and SMAD Family Member 7 (SMAD7).

**mGLS and squamous cell carcinoma**

mGLS is known to be associated with an increased risk of malignant transformation [47]. To characterize the molecular foundations for this association, we compared the mGLS gene expression profiles to those of lung squamous cell carcinoma (LUSC) from The Cancer Genome Atlas (TCGA) in the absence of an available transcriptomic dataset for penile carcinoma. We identified 17 genes that were commonly upregulated in both the mGLS dataset and in the paired samples of the TCGA-LUSC cohort (Fig. 3C). The Gene Ontology (GO) analysis of this shared gene set demonstrated an enrichment of the genes associated with cell division (“cell division”, KW-0835, P =  0.0084). Among the shared upregulated genes were G2/mitotic-specific cyclin-B1 (CCNB1) and Ribonuclease A Family Member 7 (RNASE7); their expression was elevated by a factor of 2.14 in mGLS and by a factor of 12.38 in LUSC. The gene encoding RNASE7 was also consistently upregulated by a factor of 5.17 in the mGLS cohort and by a factor of 20.97 in LUSC.

**Neoantigens and long noncoding RNAs**

A prominent hypothesis regarding the pathogenesis of autoimmune diseases is the presentation of foreign or altered self-antigens that trigger an aberrant immune response. Earlier works have identified the ECM1 protein as a potential autoantigen and showed the presence of anti-ECM1 antibodies in most patients [7]. However, subsequent studies suggested that ECM1 autoantibodies are not involved in the triggering of the onset of GLS and, instead, represent an epiphenomenon of the disease progression [48]. We, therefore, focused on characterizing pathogenic or endogenous transcripts with a potential to generate novel immunogenic peptides.

An earlier study of VLS by RNA-Seq proposed that the autoimmune response could be triggered by the hepatitis C virus (HCV) polyU/UC motif integrated into the host genome [14]. While the mechanism of such integration remains elusive for an RNA virus without reverse transcription activity, evidence exists for the presence of HCV sequences in the host DNA [49]. In revisiting this hypothesis, we performed a direct taxonomic classification of short read sources using Kraken2 [34] on both male and female GLS datasets. It revealed that viral reads constitute less than 1% of all reads with no detectable enrichment in GLS samples. Furthermore, we assessed the potential for altering protein-coding sequences by counting the genomic and exonic insertions in RNA-Seq read alignments that contain three or more consecutive uridine nucleotides. No enrichment for such insertions was observed in GLS patients, and the frequency of exonic insertions was remarkably low, approximately one hundred reads per sample (Supplementary 1, Fig. S1). Overall, our analysis does not confirm the presence of HCV-derived polyU/UC sequences in GLS transcriptomes. The original association [14] may have originated from misaligning short reads with polyA tails to viral genomes, which generates false-positive signals for polyU/UC-like motifs.

We subsequently checked endogenous sources of neoantigens among long non-coding RNAs (lncRNAs) that contain unannotated open reading frames with a potential to encode novel peptides. Differential gene expression analysis identified a total of seven lncRNAs with significant changes in the expression level (P < 0.05 and |log2 FC| > 1 in both datasets), and only two of them were upregulated in both male and female patients (Fig. 3D). One of them, the DRAIC lncRNA, was found to contain a candidate open reading frame (Fig. 3E). According to the Ribo-seq data from the GWIPS-viz portal [50], the translational potential of DRAIC showed a weak but consistent signal in its genomic locus. Another transcript commonly overexpressed in both mGLS and VLS is MMP2 Antisense RNA 1 (MMP2-AS1), which is associated with lung non-small cell cancers [51]. However, the expression levels of both transcripts were remarkably low (TPM = 0.18 and 0.78, respectively). Other noncoding RNA species didn’t show a concordant and significant change in mGLS and VLS.

**Validation of deregulated genes**

**Fig. 4:**
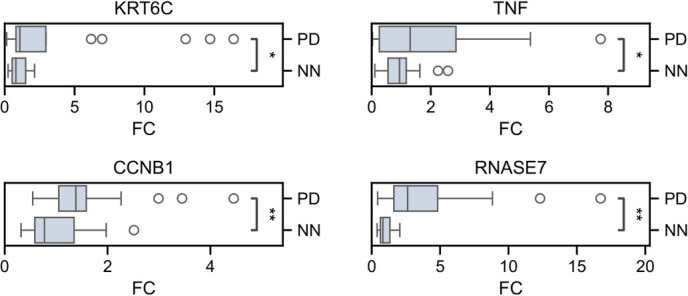
RT-qPCR validation of upregulation for Keratin 6C (KRT6C), tumor necrosis factor (TNF), Cyclin B1 (CCNB1), and Ribonuclease A Family Member 7 (RNASE7). Gene expression levels were normalized to the expression levels of GAPDH. PD – mGLS group. NN – control group. Box plots represent expression fold change (FC) relative to the mean expression in the control group. For each gene, five independent replicates were examined in each of the eight patients. Statistically significant differences at the significance levels of 5% and 10% are denoted by * and **, respectively

In order to validate the deregulation of markers predicted from the RNA-seq experiments, we performed a RT-qPCR analysis of the selected genes in five technical replicates for RNA obtained from each of the eight patients. The gene expression level measured by RT-qPCR was normalized to that of the glyceraldehyde-3-phosphate dehydrogenase (GAPDH) gene, which was used as a reference. As evidenced by FC values with respect to the median value in the control group, KRT6C, TNF, CCNB1, and RNASE7 were significantly overexpressed in mGLS samples compared to the controls, with a remarkable number of outliers presumably indicating transcriptomic heterogeneity (Fig. 4). The expression levels of VIM and TGFB1 were not significantly different between mGLS and the control groups, while the expression level of DRAIC lncRNA was at borderline detection levels in both, consistent with its low TPM counts in the RNA-seq data. The latter result does not invalidate its role in the pathogenesis of mGLS, since even low-expressed transcripts can produce immunogenic peptides, which may have a significant impact on the immune response.

## DISCUSSION

The heterogeneity of transcriptomic signatures inferred from RNA-seq experiments is often attributed to the different compositions of the cellular subtypes comprising tissue samples, although other factors, such as variation in the presentation, clinical manifestations, and severity of the disease, also play a crucial role. Histological studies have uncovered vast interpatient heterogeneity in lesional skin from VLS patients [23]. Transcriptomic heterogeneity in mGLS has also been reported, with two distinct subtypes corresponding to the activation of the immune pathways and epithelial cell proliferation pathways, respectively, while they share common signatures related to hyperkeratosis [52]. In our study, the observed heterogeneity in the transcriptomic signatures in mGLS samples can primarily be attributed to a different composition of the immune infiltrate; namely in the relative abundance of CD8^+^ T cells, T-regulatory cells, and neutrophils, which is responsible for the lack of clustering of mGLS samples in the PCA plot. More detailed estimates of GLS heterogeneity can be inferred by analyzing single-cell RNA-seq experiments (Table 1), which falls outside the scope of this short report.

Remarkably, the gene set enrichment analysis has identified the most overrepresented functional gene categories related to the integrity of epidermal cutaneous structure and innate immunity, but also to metabolic categories such as the urea cycle. The latter enrichment was driven by the upregulation of three genes including two arginases (ARG1, ARG2), which could have implications for immunity, since arginine catabolism by myeloid cells is known to contribute to modulation of the immune response [53]. However, in spite of these immune-related signals, the change in epidermal transcriptional programs was the most pronounced alteration. Functional alternations in splicing programs are harder to assess, since the exact roles of splice isoforms remain largely unknown. Nevertheless, it appears plausible that splicing changes in the DMKN gene produced by keratinocytes could reflect the shift to the DMKN-β splice isoform, in response to proinflammatory cytokines [54].

The lack of the expected upregulation of known GLS markers such as VIM and CTNNB1 in both mGLS and VLS suggests a large discrepancy between protein expression and mRNA abundance. For ECM1, the lack of deregulation aligns with the previous study, which reported this effect only in pediatric-onset cases but not in the adult-onset cohort [17]. The absence of LGALS7 deregulation, which has been implicated in fibroblast proliferation in VLS [55], may again be attributed to the differences between immunohistochemical staining without PCR confirmation in whole-tissue samples [55]. A number of genes associated with the progression of autoimmune diseases were activated in both mGLS and VLS, including CYBB, SMAD7, and RUNX3. They play distinct roles in immune regulation: CYBB is important for reactive oxygen species production in phagocytes [56], SMAD7 inhibits TGF-β signaling by promoting TGF-β degradation [57], often resulting in a pro-inflammatory effect [58], and RUNX3 also modulates TGF-β responses in dendritic cells [59]. Their upregulation supports the idea of the involvement of the immune response in GLS pathogenesis, consistent with the hypothesis of its autoimmune origin, although without any clear mechanistic model [46].

It is remarkable that two non-coding transcripts (DRAIC and MMP2-AS1) that were upregulated in both mGLS and VLS are related to cancer pathogenesis. DRAIC is known to function as a tumor suppressor in prostate and other cancers by inhibiting NF-κB signaling [60]. It has not been previously reported as a protein-coding gene; however, Ribo-seq experiments weakly indicate that DRAIC may actually be translated. The MMP2-AS1 lncRNA is known to contribute to the progression of renal cell carcinoma by modulating the miR-34c-5p/MMP2 axis [61]. Although there are no open reading frames in MMP2-AS1, antisense transcripts often encode micropeptides with important functions in cancer [62]. Genes commonly upregulated in mGLS and non-small cell cancers include CCNB1, a central regulator of the G2/M phase transition [63], which is upregulated in many types of cancer and linked to poor prognosis [64], and RNASE7, an antimicrobial protein secreted by various epithelial tissues and linked to cutaneous squamous cell carcinoma [65]. All these mRNAs could be viewed as common potential biomarkers of GLS or targeted by therapeutic modulatory approaches.

## CONCLUSIONS

Genital lichen sclerosus has long been considered an enigmatic and challenging disease. Here, we conducted a comparative survey of transcriptomic signatures, which has revealed multiple genes that are commonly deregulated in mGLS and VLS, including the previously unreported biomarkers KRT6 and CHIT1, genes with known links to GLS and autoimmune diseases (TNF, CYBB, SMAD7, and RUNX3), long non-coding RNAs DRAIC and MMP2-AS1, as well as genes commonly deregulated in GLS and in squamous cell carcinomas. These findings significantly expand the current knowledge on GLS pathogenesis and open new avenues towards its correct diagnosis and treatment.

## Abbreviations

DEGs: differentially expressed genes
RT-qPCR: quantitative reverse transcription PCR
GLS: genital lichen sclerosus
mGLS: male genital lichen sclerosus
VLS: vulvar lichen sclerosus
PCA: principal component analysis
TMP: transcripts per million

## References

[1] Latini A., Cota C., Orsini D., Cristaudo A., Tedesco M. (2018). Male and female genital lichen sclerosus. Clinical and functional classification criteria.. Postepy Dermatol Alergol..

[2] Kantere D., Löwhagen GB., Alvengren G., Månesköld A., Gillstedt M., Tunbäck P. (2014). The clinical spectrum of lichen sclerosus in male patients - a retrospective study.. Acta Derm Venereol..

[3] Virgili A., Borghi A., Toni G., Minghetti S., Corazza M. (2014). Prospective clinical and epidemiologic study of vulvar lichen sclerosus: analysis of prevalence and severity of clinical features, together with historical and demographic associations.. Dermatology..

[4] Spekreijse JJ., Streng BMM., Vermeulen RFM., Voss FO., Vermaat H., van Beurden M. (2020). The risk of developing squamous cell carcinoma in patients with anogenital lichen sclerosis: A systematic review.. Gynecol Oncol..

[5] Kirtschig G. (2016). Lichen sclerosus-presentation, diagnosis and management.. Dtsch Arztebl Int..

[6] Oyama N., Hasegawa M. (2022). Lichen sclerosus: A current landscape of autoimmune and genetic interplay.. Diagnostics (Basel)..

[7] Oyama N., Chan I., Neill SM. (2003). Autoantibodies to extracellular matrix protein 1 in lichen sclerosus.. Lancet..

[8] Carlson BC., Hofer MD., Ballek N., Yang XJ., Meeks JJ., Gonzalez CM. (2013). Protein markers of malignant potential in penile and vulvar lichen sclerosus.. J Urol..

[9] Sever M., Trčko K., Zidarič T., Maver T. (2025). Exploring genital lichen sclerosus: navigating from pathophysiology to precise diagnostic approaches.. Biomedicines..

[10] Azurdia RM., Luzzi GA., Byren I. (1999). Lichen sclerosus in adult men: a study of HLA associations and susceptibility to autoimmune disease.. Br J Dermatol..

[11] Gao XH., Barnardo MC., Winsey S. (2005). The association between HLA DR, DQ antigens, and vulval lichen sclerosus in the UK: HLA DRB112 and its associated DRB112/DQB10301/04/09/010 haplotype confers susceptibility to vulval lichen sclerosus, and HLA DRB10301/04 and its associated DRB10301/04/DQB10201/02/03 haplotype protects from vulval lichen sclerosus.. J Invest Dermatol..

[12] Torres A., Zaborek-Łyczba M., Łyczba J., Mertowska P., Mertowski S., Grywalska E. (2022). The importance of immunological disorders in the pathogenesis of lichen sclerosus in pediatric patients: A systematic review.. Int J Mol Sci..

[13] Šuler Baglama Š., Jemec GBE., Zmazek J., Trčko K. (2024). Sex-related variations in comorbidities in lichen sclerosus: A systematic review and meta-analysis.. Acta Derm Venereol..

[14] Cong Q., Guo X., Zhang S. (2021). HCV poly U/UC sequence-induced inflammation leads to metabolic disorders in vulvar lichen sclerosis.. Life Sci Alliance..

[15] Yu Z., Wang Z., Mao G. (2025). Multi-omics analysis reveals the host-microbe interactions on the dysbiosis of tissue microbiota in male genital lichen sclerosus-induced urethral strictures.. Microbiol Spectr..

[16] Lin L., Liu Y., Wang X. (2025). Multi-omics analysis unveiled fibroblast-mediated pathogenesis in male genital lichen sclerosus.. Cell Biosci..

[17] Tran DA., Tan X., Macri CJ., Goldstein AT., Fu SW. (2019). Lichen sclerosus: An autoimmunopathogenic and genomic enigma with emerging genetic and immune targets.. Int J Biol Sci..

[18] Zhang W., Zhang J., Jiao D. (2024). Single-cell RNA sequencing reveals a unique fibroblastic subset and immune disorder in lichen sclerosus urethral stricture.. J Inflamm Res..

[19] Wang J., Fan H., Bao Z., Li G., Wang L., Zhang D. (2025). Immune dysregulation and cellular composition in lichen sclerosus revealed by integrative epigenetic analysis with cell type deconvolution.. J Inflamm Res..

[20] Edmonds E., Barton G., Buisson S. (2011). Gene expression profiling in male genital lichen sclerosus.. Int J Exp Pathol..

[21] Pilatz A., Altinkilic B., Schormann E. (2013). Congenital phimosis in patients with and without lichen sclerosus: distinct expression patterns of tissue remodeling associated genes.. J Urol..

[22] Wang L., Lv Q., Guo J., Wang J., Pan J. (2022). Transcriptome profiling and network analysis provide insights into the pathogenesis of vulvar lichen sclerosus.. Front Genet..

[23] Sun P., Kraus CN., Zhao W. (2025). Spatial and single-cell transcriptomics reveal keratinocytes as key players in vulvar lichen sclerosus pathogenesis.. J Invest Dermatol..

[24] Terlou A., Santegoets LA., van der Meijden WI. (2012). An autoimmune phenotype in vulvar lichen sclerosus and lichen planus: a Th1 response and high levels of microRNA-155.. J Invest Dermatol..

[25] Chen S., Zhou Y., Chen Y., Gu J. (2018). fastp: an ultra-fast all-in-one FASTQ preprocessor.. Bioinformatics..

[26] Dobin A., Davis CA., Schlesinger F. (2013). STAR: ultrafast universal RNA-seq aligner.. Bioinformatics..

[27] Liao Y., Smyth GK., Shi W. (2014). featureCounts: an efficient general purpose program for assigning sequence reads to genomic features.. Bioinformatics..

[28] Finotello F., Mayer C., Plattner C. (2019). Molecular and pharmacological modulators of the tumor immune contexture revealed by deconvolution of RNA-seq data.. Genome Med..

[29] Sturm G., Finotello F., Petitprez F. (2019). Comprehensive evaluation of transcriptome-based cell-type quantification methods for immuno-oncology.. Bioinformatics..

[30] Muzellec B., Teleńczuk M., Cabeli V., Andreux M. (2023). PyDESeq2: a python package for bulk RNA-seq differential expression analysis.. Bioinformatics..

[31] Law CW., Chen Y., Shi W., Smyth GK. (2014). voom: Precision weights unlock linear model analysis tools for RNA-seq read counts.. Genome Biol..

[32] Sherman BT., Hao M., Qiu J. (2022). DAVID: a web server for functional enrichment analysis and functional annotation of gene lists (2021 update).. Nucleic Acids Res..

[33] Shen S., Park JW., Lu ZX. (2014). rMATS: robust and flexible detection of differential alternative splicing from replicate RNA-Seq data.. Proc Natl Acad Sci U S A..

[34] Wood DE., Lu J., Langmead B. (2019). Improved metagenomic analysis with Kraken 2.. Genome Biol..

[35] Lim L., Jonsson AH. (2025). CD8+ T cells you sshould know about in autoimmunity: Current paradigms of T cell pathogenesis in autoimmune disease.. Curr Allergy Asthma Rep..

[36] Dikiy S., Rudensky AY. (2023). Principles of regulatory T cell function.. Immunity..

[37] Fu X., Liu H., Huang G., Dai SS. (2021). The emerging role of neutrophils in autoimmune-associated disorders: effector, predictor, and therapeutic targets.. MedComm (2020)..

[38] Dennis G., Sherman BT., Hosack DA. (2003). DAVID: Database for annotation, visualization, and integrated discovery.. Genome Biol..

[39] Matsui T., Hayashi-Kisumi F., Kinoshita Y. (2004). Identification of novel keratinocytesecreted peptides dermokine-alpha/-beta and a new stratified epithelium-secreted protein gene complex on human chromosome 19q13.1.. Genomics..

[40] Utsunomiya A., Chino T., Utsunomiya N. (2020). Homeostatic function of dermokine in the skin barrier and inflammation.. J Invest Dermatol..

[41] Saini N., Acharjee A. (2025). Identifying inflammatory bowel disease subtypes: a comprehensive exploration of transcriptomic data and machine learning-based approaches.. Ther Adv Gastroenterol..

[42] Farias TDJ., Augusto DG., de Almeida RC., Malheiros D., Petzl-Erler ML. (2019). Screening the full leucocyte receptor complex genomic region revealed associations with pemphigus that might be explained by gene regulation.. Immunology..

[43] Martinelli M., Aguilar G., Lee DSM. (2022). The poly(C)-binding protein Pcbp2 is essential for CD4+ T cell activation and proliferation.. iScience..

[44] Zhang X., Yin M., Zhang LJ. (2019). Keratin 6, 16 and 17-critical barrier alarmin molecules in skin wounds and psoriasis.. Cells..

[45] Di Francesco AM., Verrecchia E., Manna S., Urbani A., Manna R. (2022). The chitinases as biomarkers in immune-mediate diseases.. Clin Chem Lab Med..

[46] Rajalingam A., Ganjiwale A. (2024). Identification of common genetic factors and immune-related pathways associating more than two autoimmune disorders: implications on risk, diagnosis, and treatment.. Genomics Inform..

[47] Nasca MR., Innocenzi D., Micali G. (1999). Penile cancer among patients with genital lichen sclerosus.. J Am Acad Dermatol..

[48] Edmonds EV., Oyama N., Chan I., Francis N., McGrath JA., Bunker CB. (2011). Extracellular matrix protein 1 autoantibodies in male genital lichen sclerosus.. Br J Dermatol..

[49] Zemer R., Kitay Cohen Y., Naftaly T., Klein A. (2008). Presence of hepatitis C virus DNA sequences in the DNA of infected patients.. Eur J Clin Invest..

[50] Tierney JAS., Świrski MI., Tjeldnes H. (2025). RiboSeq.Org: an integrated suite of resources for ribosome profiling data analysis and visualization.. Nucleic Acids Res..

[51] Rappaport N., Twik M., Plaschkes I. (2017). MalaCards: an amalgamated human disease compendium with diverse clinical and genetic annotation and structured search.. Nucleic Acids Res..

[52] Xiu X., Yu Z., Kravvas G. (2025). Molecular subtypes of balanopreputial and urethral male genital lichen sclerosus: Distinct transcriptomic and clinicopathological profiles.. Lab Invest..

[53] Rodriguez PC., Ochoa AC., Al-Khami AA. (2017). Arginine metabolism in myeloid cells shapes innate and adaptive immunity.. Front Immunol..

[54] Higashi K., Hasegawa M., Yokoyama C., Tachibana T., Mitsui S., Saito K. (2012). Dermokine-β impairs ERK signaling through direct binding to GRP78.. FEBS Lett..

[55] Zhao Y., Zhao S., Li H., Qin X., Wu X. (2018). Expression of galectin-7 in vulvar lichen sclerosus and its effect on dermal fibroblasts.. Oncol Lett..

[56] Bedard K., Krause KH. (2007). The NOX family of ROS-generating NADPH oxidases: physiology and pathophysiology.. Physiol Rev..

[57] Kavsak P., Rasmussen RK., Causing CG. (2000). Smad7 binds to Smurf2 to form an E3 ubiquitin ligase that targets the TGF beta receptor for degradation.. Mol Cell..

[58] Sanjabi S., Zenewicz LA., Kamanaka M., Flavell RA. (2009). Anti-inflammatory and proinflammatory roles of TGF-beta, IL-10, and IL-22 in immunity and autoimmunity.. Curr Opin Pharmacol..

[59] Fainaru O., Woolf E., Lotem J. (2004). Runx3 regulates mouse TGF-beta-mediated dendritic cell function and its absence results in airway inflammation.. EMBO J..

[60] Saha S., Kiran M., Kuscu C. (2020). Long noncoding RNA DRAIC inhibits prostate cancer progression by interacting with IKK to inhibit NF-κB activation.. Cancer Research.

[61] Fan B., Niu Y., Ren Z. (2022). Long noncoding RNA MMP2-AS1 contributes to progression of renal cell carcinoma by modulating miR-34c-5p/MMP2 axis.. J Oncol..

[62] Zhang T., Li Z., Li J., Peng Y. (2025). Small open reading frame-encoded microproteins in cancer: identification, biological functions and clinical significance.. Mol Cancer..

[63] Jackman M., Marcozzi C., Barbiero M. (2020). Cyclin B1-Cdk1 facilitates MAD1 release from the nuclear pore to ensure a robust spindle checkpoint.. J Cell Biol..

[64] Dai P., Xiong L., Wei Y. (2023). A pancancer analysis of the oncogenic role of cyclin B1 (CCNB1) in human tumors.. Sci Rep..

[65] Harder J., Schroder JM. (2002). RNase 7, a novel innate immune defense antimicrobial protein of healthy human skin.. J Biol Chem..

